# Dermatology

**Published:** 1899-09

**Authors:** Henry Waldo


### DERMATOLOGY.

At a debate of the Dermatological Society of London, November 9th, 1898, on **general exfoliative dermatitis or pityriasis rubra,** Dr. Walter G. Smith[1] said that in regard to nomenclature, etymology is a fallible guide in medical terminology, and he did not think it mattered much whether we choose to call it exfoliative dermatitis or pityriasis rubra, except that it would be well we should all agree, for convenience of reference, to adhere to the one designation, and the former term seems likely to prevail. Intense vivid redness he considers to be one of its most distinctive features; yet just as a zoologist finds no difficulty in acknowledging a white blackbird, so the dermatologist need not stumble at a white pityriasis rubra. Dr. Walter Smith is not aware that any important blood-changes have been made out in connection with dermatitis exfoliativa, although pseudo-leukæmia has been noted. Nor has the disease been proved to be due to a microbe, so we are left to mere conjecture. It has been suggested, especially by Mr. Hutchinson, that pityriasis rubra and other generalised forms of dermatitis are **etiologically related to the nervous system,** and may, in fact, be regarded as neuroses. In support of this view Mr. Hutchinson adduces the occurrence of joint-pains in many cases in the early stage, the occurrence of coma in one patient, and the fact that intolerable itching in the middle of the back ushered in the eruption in several cases. Hebra invokes the convenient hypothesis of vaso-motor disturbance. Dr. Crocker makes out a stronger case for association with rheumatism. He, by-the-by, prefers the term pityriasis rubra as being the original name, and thinks it better policy to stick to the old labels than to use new ones. He considered that pityriasis rubra represented a well-defined group of symptoms which might **develop primarily, or secondarily,** to some antecedent form of dermatitis, and that when the secondary cases were fully developed they were practically identical in their symptomology and course with the primary cases. The secondary cases might supervene not only upon almost any form of dermatitis, such as psoriasis, eczema, seborrhœic eczema, and lichen planus, in that order of frequency, but also, after the application of irritants to the skin, such as

[1] *Brit. J. Dermat.*, 1898, x. 437.

chrysarobin, arnica, and mercury. In the early days of the century, when mercurial inunction was used in a wholesale manner, this complication was not very rare. Clinically, many of the cases of the epidemic form described by Savill were indistinguishable from the non-contagious and classical form, and it showed that the presence of a special microbe was capable of producing the disease in some cases. Dr. Crocker had seen two cases in association with insanity, and he asked the members of the Society if they had had a corresponding experience. Dr. Walter Smith said that if dermatitis exfoliativa is recognised as a primary affection, and also as following upon such different affections as eczema, psoriasis, erythemata, lichen ruber, erysipelas, dermatitis herpetiformis, pemphigus, and perhaps, in children, syphilis, and if, further, it is allowed that this condition may be started by strong external irritants, and perhaps by internal remedies, *e.g.* chloralamide,[1] then in the absence of any uniform etiological factor, it is more rational to consider it as an accidental phenomenon, or as an evolution of some other affection, rather than an independent entity. Pityriasis rubra has been observed to supervene upon psoriasis, and when the pityriasis rubra got well the psoriasis resumed its normal course. As to frequency of primary and secondary cases of exfoliative dermatitis, Stephen Mackenzie's statistics give a nearly equal frequency (eleven primary and ten secondary). Moreover, although the first attack may be plainly secondary to some existing cause, yet it may be impossible to discover any adequate cause for subsequent recurrences. Dr. Smith considers that no certain boundary line can as yet be drawn between acute general eczema—possibly, also psoriasis—and exfoliative dermatitis. Dr. Galloway believes the secondary forms of the disease most commonly succeed psoriasis, and he considered that there was some danger in producing this grave secondary state by over-medication. He thought the histological alterations were distinctive, although they did not throw much light on the pathology of the disease, except to emphasise, what was pretty clear from clinical observation, that the poison which caused the disease was from within and not from without. Dr. J. J. Pringle's remarks pointed strongly towards the probability of the existence of **changes in the central nervous system,** possibly of toxæmic origin, as the determining factor in the production of general exfoliative dermatitis. These were: (1) the frequently neurotic character of the persons attacked; (2) the rapidity with which the condition develops and generalises itself; (3) the prominence of extreme itching, insomnia, and insanity among the leading symptoms; (4) the marked susceptibility to changes of temperature, and the instability of the peripheral circulation; (5) the remarkable changes occurring in the skin appendages—hair and nails. He considered that the prominent

[1] Pye-Smith, *Guy's Hosp. Rep.*, 1877, 3rd Ser., xxii. 151; 1881, 3rd Ser., xxv. 205.

*rôle* played by alcohol, at all events as a predisposing cause, had been generally underestimated, and that the existence of **albuminuria** always constituted a grave complication. As to treatment, arsenic was deleterious in the early stages of the disease, and he suspected that it was often responsible for its supervention upon cases of psoriasis. Dr. Leslie Roberts thought that the process of dermatitis exfoliativa was not purely ectodermal, but rather a neuro-ectodermal process in which the sensitive matter of the cerebro-spinal axis operated with or upon the epithelium and underlying vessels and nerves. He had come to this conclusion after careful consideration of certain clinical and histological facts; to wit, the almost startling suddenness with which the entire epidermis became altered in character, the predominance of nervous complications in the course of the disease, and the very characteristic inward growths of the epithelial cones. He considered that the morbid epithelial changes in dermatitis exfoliativa were certainly under some powerful nerve-influence; but he could not say, in the light of present evidence, how this influence occurs. Mr. Malcolm Morris thought the disease was peculiar for the reason that, in spite of the great redness and desquamation present, there was an entire absence of thickening; indeed there seemed to be rather an atrophy of the corium present, as shown by a slight thinning of the skin when picked up between the fingers. He considered that the presence of albumin in the urine was a factor justifying the utmost caution in regard to prognosis, and that in his experience such cases were often fatal.[1]

**Pityriasis rosea**, though a comparatively rare disease, occurs more frequently than is generally supposed, for cases are often overlooked or incorrectly diagnosed. A typical case runs a very clear and definite course. Without any previous symptoms, there appears on the trunk, somewhere in the region of the waist, as the "herald" of the disease, a reddish-yellow spot which expands into a patch, circular in form, very little elevated, with a rosy-red border and a yellow centre. The shade of yellow which forms the centre is sometimes described as fawn-coloured; frequently it very closely resembles the colour of chamois leather. Often enough this patch is entirely overlooked, and the first the patient knows of the disease is about a week later, when the whole trunk is covered with a profuse eruption of spots similar in nature to, though smaller in size than, the original one. All do not expand into circles; many remain as spots, and to this variety of the disease the name pityriasis rosea **maculata** is applied. When they do expand into circles, the adjective **circinata** is employed instead. Too much stress must not be placed on the identification of the "herald"

[1] *Brit. J. Dermat.*, 1898, x. 463.

patch; in the majority of cases it is not discoverable. The eruption is almost limited to the trunk. A few spots may be found about the shoulders, and a few on the thighs; but it is rare on the face and on the distal ends of the limbs, though it may be limited to them. In spite of the name, there is not often much scaliness. After a duration of from five to eight weeks the eruption gradually disappears. The cause of the disease is unknown. No organisms have been found which could be definitely associated with it. Dr. Norman Walker gives[1] a picture which is an admirable illustration of this disease.

The numerous reports from foreign sources which ascribe to the **X-ray therapeutic properties**, and give the results produced in cases of cancer and lupus vulgaris, and also claim for it a retarding action upon the growth of bacteriological cultures, have led Dr. Charles Leonard to study the electrical conditions present in order to determine whether the results obtained cannot be more reasonably explained by the action of physical and physiological forces the properties of which are perfectly known and understood. His conclusions are that the X-ray "burn" is not the result of the action of the X-ray, but the dermatitis produced is the result of the static currents or charges induced in the tissues by the high-potential induction field surrounding the X-ray tube. The therapeutic properties attributed to the X-ray do not belong to it, but are due to the static charges and currents induced in the tissues, which have long been known to be capable of producing similar results.

The dermatitis, which has been called an X-ray "burn," is the result of an interference with the nutrition of the part by the induced static charges. The patient may be absolutely protected from the harmful effects of this static charge by the interposition between the tube and the patient of a grounded sheet of conducting material that is readily penetrable by the X-ray; a thin sheet of aluminium or gold-leaf spread upon cardboard is an effectual shield.[2]

Dr. S. Pollitzer, of New York, says[3] that the peculiar yellow plaques and nodules in the skin known as **xanthoma** have been the subject of extensive studies on the part of pathologists and dermatologists, ever since they were first described by Addison and Gull in 1850. The greatest diversity of opinion exists as to their nature. The clinical grounds for separating xanthoma of the eyelids from multiple xanthoma are as follows: The nodules of xanthoma multiplex are firm, round, elevated papules; the patches of eyelid xanthoma are soft plaques on the level of the skin. Eyelid xanthoma persists through life; multiple xanthoma sooner or later undergoes involution. Eyelid xanthoma is quite common; multiple xanthoma is extremely rare. If the eyelids were in this preponderating degree the seat of predilection for a common xanthoma, we should expect to

[1] *An Introduction to Dermatology*, 1899. [2] *N. York M. J.*, 1898, lxviii. 18.
[3] *Tr. Am. Dermat. Ass.*, 1897, 21.

find the eyelids affected in every case of multiple xanthoma; but, as a matter of fact, the two forms are rarely associated in the same individual. The author has been able to show that common eyelid xanthoma is not a new growth, but is due to a degeneration of pre-existing embryonally misplaced muscular tissue. The so-called xanthoma cell is a fragmented muscle fibre in a state of granulo-fatty degeneration, with proliferation of the muscle-nuclei. This explanation of the origin of eyelid xanthoma harmonises with a number of hitherto unexplained clinical and pathological facts, *e.g.* the absence of any clinical signs of tumour; its almost exclusive occurrence in the face, where peculiar muscular conditions prevail; its heredity; its usual development after middle age, when degenerative processes are apt to occur; the peculiar yellow pigment that is always present in muscles undergoing fatty degeneration, etc. The structure of multiple xanthoma is shown to be wholly different from that of eyelid xanthoma. It forms a sharply circumscribed tumour in the cutis. It is an irritative hyperplastic development of connective tissue, whose cells produce fibrous tissue on the one hand, or undergo fatty degeneration on the other. In diabetic xanthoma the process is a little more diffuse and the tendency towards fatty degeneration more marked. In both, irregular patches of granulo-fatty matter, interspersed with cellular detritus, occur in the middle of the nodules as the results of the fatty degeneration of the cells. In over 85 per cent. of the recorded cases of multiple xanthoma, far too large a number to be accounted a mere chance, there was either diabetes or some severe lesion of the liver with jaundice. The author thinks it likely that research may show that the fibrous nodes and fusiform enlargements of tendons in chronic rheumatism are to be placed in the same general class as the nodes of xanthoma. We should then have a large group of diseases, hepatic, diabetic, rheumatic, all characterised by toxæmic conditions, in all of which irritative connective-tissue lesions occur in the skin and elsewhere. At one end of this series we should have the persistent fibrous node of rheumatism; at the other, the transient nodules of diabetic xanthoma, while between them, intermediate in its tendency toward the formation of fibrous tissue and fatty degeneration, ultimately undergoing involution, would stand the nodule of common multiple xanthoma.

HENRY WALDO.

